# Trojan Horse Delivery Strategies of Natural Medicine Monomers: Challenges and Limitations in Improving Brain Targeting

**DOI:** 10.3390/pharmaceutics17030280

**Published:** 2025-02-20

**Authors:** Kelu Lei, Lanyu Zhou, Min Dan, Fei Yang, Tiantian Jian, Juan Xin, Zhigang Yu, Yue Wang

**Affiliations:** 1 Department of Pharmacy, Ya’an People’s Hospital-West China Ya’an Hospital, Sichuan University, Ya’an 625000, China; 2020ks210@stu.cdutcm.edu.cn (K.L.); huanxigungun@163.com (M.D.); yf1564802295@163.com (F.Y.); 15182376356@163.com (T.J.); xinjuan1123@163.com (J.X.); 2 State Key Laboratory of Southwestern Chinese Medicine Resources, School of Pharmacy, Chengdu University of Traditional Chinese Medicine, Chengdu 611137, China; 2020ks360@stu.cdutcm.edu.cn; 3 State Key Laboratory for Quality Ensurance and Sustainable Use of Dao-Di Herbs, National Resource Center for Chinese Materia Medica, China Academy of Chinese Medical Sciences, Beijing 100700, China

**Keywords:** central nervous system diseases, blood–brain barrier, natural products, trojan horse strategy, nanotechnology, drug delivery, brain targeting

## Abstract

Central nervous system (CNS) diseases, such as brain tumors, Alzheimer’s disease, and Parkinson’s disease, significantly impact patients’ quality of life and impose substantial economic burdens on society. The blood–brain barrier (BBB) limits the effective delivery of most therapeutic drugs, especially natural products, despite their potential therapeutic effects. The Trojan Horse strategy, using nanotechnology to disguise drugs as “cargo”, enables them to bypass the BBB, enhancing targeting and therapeutic efficacy. This review explores the applications of natural products in the treatment of CNS diseases, discusses the challenges posed by the BBB, and analyzes the advantages and limitations of the Trojan Horse strategy. Despite the existing technical challenges, future research is expected to enhance the application of natural drugs in CNS treatment by integrating nanotechnology, improving delivery mechanisms, and optimizing targeting characteristics.

## 1. Introduction

Central nervous system (CNS) diseases encompass diverse conditions, including brain tumors, neurodegenerative disorders (e.g., Alzheimer’s and Parkinson’s diseases), cerebrovascular diseases (e.g., stroke), and neuropsychiatric disorders (e.g., depression and epilepsy) [1]. These conditions are among the leading causes of disability and mortality globally. These diseases not only diminish the quality of life for affected individuals but also impose considerable economic burdens on society and healthcare systems [2,3]. Despite significant advancements in modern medicine, the effective delivery of many therapeutic drugs is still significantly impeded by barriers like the blood–brain barrier (BBB), blood–cerebrospinal fluid barrier (BCSFB) and blood–brain tumor barrier (BBTB) [4].

Natural products hold a significant potential in treating CNS diseases due to their diverse bioactive compounds, including polyphenols, flavonoids, and alkaloids [5,6]. These compounds exhibit a wide range of therapeutic effects, such as neuroprotection, anti-inflammation, antioxidation, and anti-apoptosis [7,8,9]. However, their brain-targeted delivery efficiency is often inadequate, limiting their clinical applications. Many natural products suffer from a low systemic bioavailability, and the BBB further restricts their access to target sites, reducing their therapeutic potential [10,11].

Recently, the Trojan Horse strategy has emerged as an innovative drug delivery approach, employing nanotechnology-based carriers to mimic natural transport mechanisms [12,13,14]. This method provides a promising solution for enhancing the brain-targeted delivery of natural drugs. The central concept of this strategy involves camouflaging drugs as “cargo” to facilitate their uptake by cells or tissues, ensuring a safe and effective delivery to target sites while circumventing rejection or obstruction by the body’s barriers and defense mechanisms [15,16]. In addition to improving drug targeting, the Trojan Horse strategy holds significant potential for enhancing therapeutic efficacy [13,14,15].

The drug delivery in the Trojan Horse strategy typically employs mechanisms such as carrier-mediated transport (CMT), receptor-mediated transcytosis (RMT), adsorption-mediated transcytosis (AMT), and other emerging delivery pathways [4,14,17]. These mechanisms enhance drug penetration across the BBB via various biological pathways and transport systems, thereby enabling a more efficient delivery of natural products to brain tissue [4,18,19].

One of the most significant advantages of the Trojan Horse strategy is its ability to target specific pathological sites, such as damaged neurons or inflamed tissues, by designing carriers that are recognized by the body’s immune system as “self”. This specificity contrasts with conventional drug delivery technologies commonly used in nuclear medicine, which lack this level of targeted interaction. Moreover, the Trojan Horse approach utilizes carriers, such as cell-penetrating peptides (CPPs) and biomacromolecules, which demonstrate a low toxicity, high specificity, and non-immunogenic properties. These carriers work by bypassing physiological barriers and delivering the therapeutic agent directly to the desired site, ensuring effective treatment while minimizing side effects.

Despite its promise, the Trojan Horse strategy faces several challenges and limitations in practical application [16,17]. For example, challenges related to selecting appropriate nanocarriers, controlling drug release, ensuring selectivity for target cells, and managing potential immune responses necessitate further research [17,20,21,22]. Furthermore, the inherent complexity of natural products and their interactions with carriers could affect the overall therapeutic efficacy [23,24].

This review provides an overview of the application of natural products in treating brain diseases, assesses the major challenges in brain-targeted delivery, and thoroughly examines the use and challenges of the Trojan Horse strategy in drug delivery. Additionally, it explores how to optimize the brain-targeted delivery of natural products by integrating modern nanotechnology, offering a structured approach and outlining future research directions. Through comprehensive analysis, this review aims to provide novel insights and inspiration for advancing the application of natural products in treating CNS diseases.

## 2. Major Factors Limiting Brain Targeting

**Figure 1:**
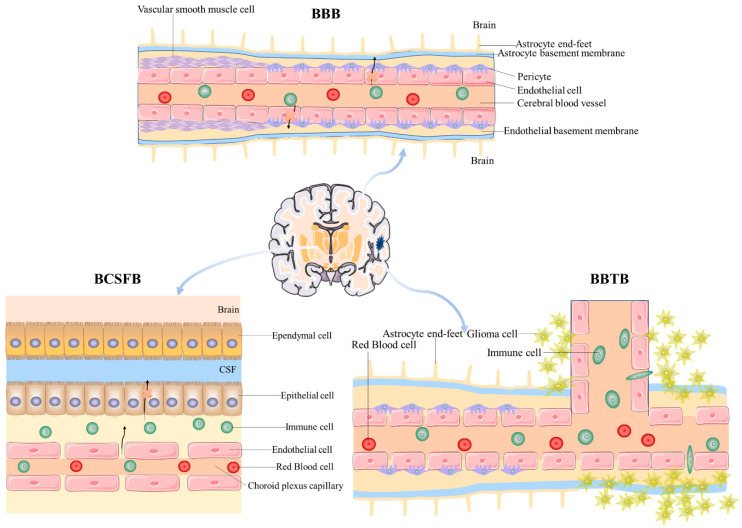
The main barriers to brain delivery. The illustration depicts the three principal barriers that regulate substance delivery to the brain: the blood–brain barrier (BBB), the blood–cerebrospinal fluid barrier (BCSFB), and the blood–brain tumor barrier (BBTB). The BBB is characterized by tightly connected endothelial cells, pericytes, and astrocyte end-feet, forming a protective interface between the cerebral vasculature and the brain parenchyma. The BCSFB is located at the choroid plexus and consists of epithelial cells that regulate the exchange between the blood and cerebrospinal fluid (CSF). The BBTB demonstrates alterations in the barrier properties due to tumor presence, affecting the permeability and interaction with the surrounding brain tissue.

Brain drug delivery is limited by multiple barriers, including the classical BBB, blood–cerebrospinal fluid barrier (BCSFB), blood–brain tumor barrier (BBTB), and other factors such as dynamic changes in intracerebral microcirculation, the complex structure of the brain interstitial barrier, narrow synaptic spaces, the impact of lymphatic excretion pathways, and the influence of inflammatory responses on barriers in pathological states [25,26]. These factors collectively act to significantly limit the effective delivery of drugs into brain tissues and their therapeutic efficacy, as shown in Figure 1. It is important to note that Figure 1 provides a simplified schematic representation of the brain’s barriers for illustrative purposes. In reality, the mechanisms of substance penetration across these barriers involve intricate and dynamic interactions between endothelial cells, tight junction proteins, transporters, and other cellular and non-cellular components, which are not fully depicted in the figure.

### 2.1. Blood–Brain Barrier

The BBB is a major obstacle to brain drug delivery, consisting of tightly connected brain endothelial cells, the basement membrane, pericytes, and astrocytes, forming a highly selective biological barrier that protects the brain from harmful substances while limiting the entry of drugs and therapeutic agents [27,28,29]. The BBB strictly controls the transport of nutrients and oxygen while preventing harmful substances from entering the brain, resulting in over 98% of small molecule drugs and nearly 100% of large molecule drugs failing to effectively penetrate [27,28,30]. Its endothelial cells restrict the passage of large molecules and water-soluble drugs through tight junctions, with drugs typically over 400 Da in molecular weight being difficult to penetrate [31,32,33,34]. Additionally, the endothelial cell membranes of the BBB express multiple efflux transporters, such as P-glycoprotein, which actively expels drugs entering the endothelial cells, reducing their concentration in the brain [35,36,37,38]. Although certain transport proteins are responsible for transporting endogenous nutrients, most exogenous drugs lack corresponding transport mechanisms, thus limiting their ability to enter the brain [27,38,39]. This strict selectivity results in many drugs having low concentrations in the brain, insufficient to reach the levels required for effective treatment, further limiting their application [30,38,40,41].

### 2.2. Blood–Cerebrospinal Fluid Barrier

The blood–cerebrospinal fluid barrier (BCSFB), composed of choroid plexus epithelial cells, serves as a crucial barrier between blood and cerebrospinal fluid [42]. Its main function is to regulate the exchange of substances through selective transport mechanisms, ensuring cerebrospinal fluid stability to provide an ideal microenvironment for the central nervous system [42,43,44,45]. The BCSFB effectively blocks most hydrophilic molecules and macromolecular compounds from entering the cerebrospinal fluid, thus protecting brain tissue from potentially harmful substances [44,45,46,47]. Within the barrier, there are various efflux transport proteins, such as P-glycoprotein and multidrug resistance-associated proteins (MRP), which can actively expel drugs from the cerebrospinal fluid [44,48,49]. Although this function protects brain tissue to some extent, it also limits the effective delivery of drugs [44,49]. Even if drugs successfully pass through the BCSFB into the cerebrospinal fluid, their distribution between the ventricular system and brain tissue may still be limited by fluid dynamics, leading to an insufficient drug concentration in target areas [44,50,51]. Compared to the BBB, the BCSFB exhibits a certain level of leakage, allowing molecules that cannot cross the BBB to enter the cerebrospinal fluid via the choroid plexus [42,52]. Drugs can transfer from the cerebrospinal fluid to the brain through three pathways: entering the blood from the cerebrospinal fluid and crossing the BBB, entering the brain through the ependyma of cerebrospinal fluid pathways, or traversing cerebrospinal fluid along perivascular spaces [53,54,55,56]. However, the minimal flow of cerebrospinal fluid within the brain parenchyma limits its penetration from perivascular spaces into the brain parenchyma [57,58]. Therefore, developing drugs or drug carriers capable of effectively crossing the BCSFB is a key direction in current research.

### 2.3. Blood–Brain Tumor Barrier

The blood–brain tumor barrier (BBTB) is a unique barrier structure in brain tumor tissue, differing from the normal blood–brain barrier [59]. Although the BBTB is generally more permeable, allowing certain drugs to pass through, it still retains some characteristics of the blood–brain barrier, limiting effective drug delivery [60,61]. In some cases, tumor growth may lead to a localized disruption of the blood–brain barrier, increasing the heterogeneity of barrier function and resulting in uneven drug delivery [62,63,64]. Additionally, features of the tumor microenvironment, such as the high-pressure interstitial conditions and vascular abnormalities, may further affect the drug distribution within the tumor [65,66,67]. These factors not only impact drug concentration but may also lead to treatment failure. In brain tumor patients, the tight junctions of the BBB become enlarged, and capillary leakage occurs, forming the BBTB [68,69]. The BBTB consists of three types of microvessels: continuous non-fenestrated microvessels, continuous fenestrated microvessels, and capillaries with endothelial channels approximately 1 μm in diameter [68,70]. The presence of the BBTB greatly limits the penetration and accumulation of drugs within the tumor [25,71]. Therefore, overcoming the BBTB to enhance the penetration capability of chemotherapeutic drugs into malignant brain tumors is a key focus for researchers. Developing novel drug delivery systems that target the characteristics of the blood–brain tumor barrier, especially drug carriers that can specifically target the tumor microenvironment, has become an important direction in current research [25,72].

In summary, brain drug delivery faces multiple barriers, which not only protect the brain from harmful substances but also significantly limit effective drug delivery. Therefore, developing drug delivery systems capable of effectively crossing these barriers is a major direction in current research.

## 3. The Application of Natural Products in the Treatment of the CNS

In the research of central nervous system diseases, natural products are gradually demonstrating unique therapeutic potential, especially in the fields of Alzheimer’s disease, Parkinson’s disease, stroke, and glioma. A detailed summary of these diseases and their related research will be provided below.

### 3.1. Alzheimer’s Disease

Alzheimer’s disease (AD) is a progressive neurodegenerative disorder characterized by a gradual decline in cognitive abilities, particularly memory loss, cognitive impairment, and behavioral abnormalities [73,74]. This disease is often accompanied by neuronal damage and β-amyloid protein deposition, leading to a significant decline in the quality of life for patients [75,76,77]. Although current drug therapies can help alleviate symptoms, their efficacy is limited and they may be accompanied by significant side effects [78,79]. Therefore, the research and application of natural medicines are gaining attention, as they show promise not only in alleviating symptoms but also in potentially slowing the disease progression [80,81].

In AD research, various natural products have shown significant efficacy. For example, Sodium oligomannate (GV-971) is a Class 1 new drug derived from marine brown algae [82]. It improves cognitive function by modulating lactobacillus abundance, inhibiting phenylalanine/isoleucine accumulation, and reducing inflammatory responses, making it a key drug in clinical treatment [83,84]. Ginkgo biloba extract can effectively improve cognitive abilities through antioxidant action and the inhibition of Aβ protein production, particularly showing positive effects in patients with mild AD and vascular dementia [85,86,87,88,89,90,91]. Papaya powder extract has shown significant antioxidant effects, reducing the production of reactive oxygen species and nitric oxide [92,93]. Similarly, sage extract and coconut oil improve cognitive function in patients through anti-inflammatory and antioxidant mechanisms, as well as reducing Aβ deposition [94,95,96,97].

Additionally, other plant components also exhibit unique therapeutic mechanisms. For example, ginsenosides reduce Aβ formation by inhibiting β- and γ-secretase activity and activating non-amyloidogenic pathways, exhibiting significant neuroprotective effects [98,99,100,101,102]. Tanshinones, due to their anti-inflammatory and antioxidant properties, have a potential value in protecting neurons, regulating cerebral blood flow, and enhancing the activity of nerve growth factors [103,104,105,106]. Cornel iridoid glycosides and polysaccharides can reduce Tau protein hyperphosphorylation, protect microtubule structure and cell morphology integrity, and improve learning and memory abilities [107]. Polygonum multiflorum polyphenols exert neuroprotective effects by regulating Tau protein dephosphorylation, reducing Aβ production, and improving brain neurotransmitter activity [108,109]. Flavonoids, polysaccharides, and lignans in Epimedium can inhibit the secretion of pro-inflammatory factors, improve mitochondrial function in the brains of AD mice, and suppress apoptosis [110]. Quercetin significantly improves the cognitive ability and anxiety in AD model mice by reducing Aβ deposition and neurofibrillary tangles in the hippocampus and amygdala regions [111,112]. Resveratrol regulates oxidative stress and anti-inflammatory responses by activating the Sirt1 pathway and inhibits Aβ peptide aggregation, showing significant effects on AD neuroinflammation and neuronal apoptosis when combined with exercise therapy [113,114]. Curcumin reduces apoptosis through multiple pathways, including the inhibition of NMDA receptor-mediated calcium ion increase and ROS production [115].

Additionally, some natural products show great promise in AD treatment. Triptolide exhibits neuroprotective effects in AD model mice by inhibiting the release of pro-inflammatory factors [116]. Plumbagin can activate the Nrf2/ARE pathway, improving cognitive deficits [117]. Embelin significantly enhances anti-amnesic effects by upregulating the expression of the brain-derived neurotrophic factor (BDNF), cAMP response element-binding protein (CREB1), and antioxidant genes (such as SOD1 and CAT) [118].

These natural compounds act through various mechanisms, including reducing neuroinflammation, inhibiting Aβ aggregation, decreasing Tau protein phosphorylation, and enhancing antioxidant capacity, providing multiple potential effective approaches for AD treatment. Their research not only provides new directions for AD treatment but also lays the foundation for developing novel drugs.

### 3.2. Parkinson’s Disease

In the study of Parkinson’s disease (PD), natural medicines also show promising therapeutic effects. PD is primarily characterized by motor dysfunction, presenting as tremors, rigidity, and bradykinesia, with its main pathological feature being the gradual loss of dopaminergic neurons in the substantia nigra of the midbrain [119,120]. Traditional treatments often rely on dopamine replacement therapy (such as levodopa), which can alleviate symptoms but often leads to drug resistance and severe side effects with long-term use [121]. In contrast, natural medicines, with their multi-mechanism and multi-target modes of action and relative safety, have become an attractive complementary or alternative treatment option [122].

Baicalein, an active compound extracted from the dried roots of the Lamiaceae plant *Scutellaria*, exhibits anti-inflammatory, antioxidant, and mitochondrial protective pharmacological effects [123]. In the rotenone-induced Parkinson’s model, baicalein alleviates neurotoxicity by inducing autophagy and restoring mitochondrial function, thus enhancing neuronal viability and improving both motor and non-motor symptoms in Parkinson’s patients [124]. Naringenin, a citrus flavanone widely found in the peels of citrus fruits, has anti-inflammatory and antioxidant effects [125]. In the MPTP-induced Parkinson’s model, naringenin enhances glutathione reductase and catalase activity, significantly improving motor function and reversing MPTP’s toxic effects [126]. Icariin, a flavonoid compound in Epimedium, provides neuroprotection by reducing microglial activation, thus mitigating dopaminergic neuron loss and motor behavioral abnormalities in LPS/6-OHDA-induced Parkinson’s models [127]. Additionally, nobiletin demonstrates neuroprotective effects in Parkinson’s models, potentially related to its regulation of oxidative stress and inflammatory responses [128]. Berberine, a multifunctional alkaloid, exhibits antioxidant, anti-inflammatory, and mitochondrial protective activities, significantly improving Parkinson’s symptoms by inhibiting oxidative stress and reducing neuroinflammation [129,130].

In PD research, besides flavonoid and alkaloid compounds, other substances like physostigmine, curcumin, and resveratrol also show significant therapeutic potential [131,132,133]. These natural compounds exhibit great potential in PD treatment through various mechanisms such as anti-inflammatory, antioxidant, mitochondrial protection, and neurotransmitter regulation [131,133]. These studies provide important clues for novel therapeutic strategies for PD, but further pharmacological research and clinical trials are needed to verify the long-term efficacy, safety, and specific application value of these natural medicines in Parkinson’s disease treatment.

### 3.3. Stroke

Stroke is an acute neurological injury caused by the disruption or insufficiency of blood supply to the brain, classified into ischemic and hemorrhagic types [134]. Patients typically require both acute treatment and rehabilitation during the recovery phase [134]. Natural medicines show promising potential in the treatment and rehabilitation of stroke, primarily by enhancing blood circulation, inhibiting inflammation, and promoting neuroregeneration [135].

Flavonoids play a significant role in stroke treatment. Puerarin, an isoflavonoid from *kudzu*, dilates cerebral vessels, boosts blood flow, reduces platelet aggregation, and enhances microcirculation, thus preventing thrombus formation [136]. Additionally, puerarin protects the vascular endothelium and aids in repairing damage, which significantly supports the recovery in patients with cerebral infarction [137]. Quercetin, with its antioxidant and anti-inflammatory activities, exhibits neuroprotective effects in ischemic stroke, significantly reducing brain damage [138]. Epigallocatechin-3-gallate (EGCG), a natural polyphenolic compound, also shows a great potential in ischemic stroke therapy, owing to its antioxidant and anti-inflammatory properties [139].

Saponin compounds have also attracted attention in stroke treatment. Panax notoginseng saponins (the active ingredient in Xuesaitong soft capsules) exhibit significant neuroprotective effects [140]. A multicenter, double-blind, placebo-controlled randomized clinical trial showed that Panax notoginseng saponins can improve the functional independence in ischemic stroke patients at 3 months [141]. Moreover, they exert anti-ischemic effects by inhibiting the TLR4/NF-κB signaling pathway and the release of inflammatory cytokines [142]. Ginsenosides, particularly ginsenoside Rg1, derived from *Panax* species, promote angiogenesis after cerebral ischemia, enhance cerebral blood flow, reduce brain injury, and significantly improve stroke prognosis [143,144].

Other natural compounds also show potential in stroke treatment. Resveratrol, with its antioxidant and anti-inflammatory activities, can reduce brain damage from ischemic stroke and provide neuroprotection [145]. Betulinic amine, an amine derivative modified from betulinic acid, has nanoparticles that can rapidly release drugs in the acidic microenvironment of a stroke. This is further enhanced by the surface modification with the CXCR4 antagonist AMD3100, which improves the targeting ability to the stroke-affected area [146]. Additionally, the efficacy in stroke treatment is further enhanced when neuroprotective peptide NA1 is encapsulated [146]. The unique advantages of natural medicines provide new insights for stroke treatment and rehabilitation, becoming an important direction in neurological disease research.

### 3.4. Glioma

Glioma is a highly invasive malignant brain tumor originating from glial cells, with a limited effectiveness of traditional therapeutic methods [147]. In the study of natural medicines, many compounds exhibit significant antitumor effects. They inhibit glioma cell proliferation and migration, induce apoptosis, and offer new possibilities for glioma treatment [148]. Curcumin is a natural compound extracted from the plant turmeric of the ginger family. It has garnered significant attention due to its various biological activities, including antioxidant, anti-inflammatory, and antitumor effects [149]. Research indicates that curcumin plays an active role in treatment by inhibiting glioma cell proliferation and migration, inducing apoptosis, and reducing tumor angiogenesis [150]. Similarly, resveratrol, found in grape skins and peanuts, demonstrates antitumor effects by modulating the cell cycle, inhibiting proliferation, and inducing apoptosis [151]. Indirubin derivatives, active components of traditional Chinese medicine, effectively inhibit glioblastoma growth and prolong survival in mice by reducing IDO1 expression [152]. Gambogic amide crosses the blood–brain barrier, accumulates in tumor regions, and targets the WDR1 protein, inhibiting glioma growth and inducing apoptosis [153]. Chlorogenic acid exerts antitumor activity by targeting mitochondrial acetyl-CoA acetyltransferase 1, currently in clinical trials for advanced malignant glioma [154]. In addition, natural peptides have also shown great promise in the treatment of gliomas. Chlorotoxin, a natural peptide extracted from scorpions, has a unique targeting effect on brain gliomas [155]. Research indicates that chlorotoxin can specifically bind to the surface of glioma cells, inhibit the proliferation and migration of tumor cells, induce apoptosis, and reduce angiogenesis [156].

In summary, natural medicines show promising applications in treating various brain diseases. Although research results are encouraging, larger clinical trials are needed to verify the efficacy and safety of these natural medicines. Future research should focus on the mechanisms of these natural compounds and their combined use with existing treatments to provide more effective therapeutic options. As research into natural medicines deepens, more effective components are expected to be discovered, opening new directions for brain disease treatment.

## 4. Application of the Trojan Horse Strategy in Drug Delivery to the Central Nervous System

**Figure 2:**
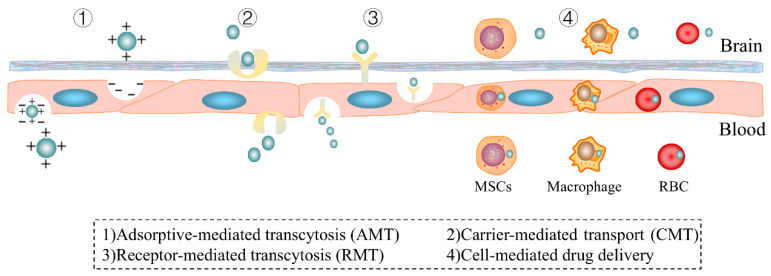
Primary pathways for Trojan Horse strategy delivery across the blood–brain barrier. The diagram illustrates four principal delivery mechanisms employed in the Trojan Horse approach: (1) adsorptive-mediated transcytosis (AMT), which involves the electrostatic interaction between positively charged molecules and the negatively charged endothelial cell surface; (2) carrier-mediated transport (CMT), which utilizes specific transport proteins to facilitate the passage of small molecules across the barrier; (3) receptor-mediated transcytosis (RMT), which exploits specific receptors on the endothelial surface to transport larger molecules or complexes; and (4) cell-mediated drug delivery, which involves the use of cells such as mesenchymal stem cells (MSCs), red blood cells (RBCs), or macrophages to transport therapeutic agents across the barrier.

The Trojan Horse strategy is an innovative method that uses naturally or artificially designed molecular mechanisms to effectively deliver drugs to the CNS [13]. Because the BBB is highly selective and restrictive to drug molecules, most systemically administered drugs have difficulty penetrating the brain. The Trojan Horse strategy offers an effective solution by “deceiving” the natural barrier mechanisms to facilitate a drug crossing of the BBB. Below are the primary delivery mechanisms of this strategy and its applications and advantages in brain drug delivery, as shown in Figure 2.

### 4.1. Receptor-Mediated Transcytosis

Receptor-mediated transcytosis (RMT) is a specialized cellular membrane transport mechanism that involves the binding of drugs or biomolecules to specific receptors on the surface of endothelial cells in the BBB, such as the transferrin receptor (TfR) and low-density lipoprotein receptor-related protein (LRP) [157]. This process disguises the drugs as natural molecules, allowing them to cleverly “sneak” into the central nervous system. RMT encompasses four key steps: receptor binding, endocytosis, intracellular transport, and exocytosis, enabling drugs to overcome the BBB’s highly selective barrier and achieve a precise delivery to the brain [158]. Like a Trojan Horse, drugs or carriers “disguise” themselves as natural molecules to evade rejection by the barrier system, thus achieving penetration and targeted delivery. This technique holds a significant potential for applications in BBB penetration, targeted drug delivery, and transport across tissue barriers. However, it still faces challenges such as receptor saturation and non-specific binding, necessitating further optimization to enhance delivery efficiency and precision [159]. The application of natural drug monomers via receptor-mediated transcytosis is summarized in Table 1.

#### 4.1.1. Transferrin Receptor

The transferrin receptor (TfR) is highly expressed in the capillary endothelial cells of the BBB and various brain tumors, making it a significant target for receptor-mediated delivery systems [190,191,192]. TfR facilitates the transport of bound drugs or carriers into brain tissue through endocytosis, with important applications in the delivery of antitumor and neurodegenerative disease drugs [191,193].

In treating brain gliomas, the application of TfR-targeted nanocarriers has shown continuous progress. For instance, Song et al. developed vincristine–tetrandrine liposomes modified with transferrin (Tf), overcoming multiple barriers in brain glioma treatment, including the BBB and multidrug resistance (MDR) [161]. These liposomes achieved an efficient drug delivery through TfR-mediated endocytosis, inhibiting tumor cell invasion and inducing cancer cell apoptosis, significantly enhancing the drug’s ability to cross the BBB. Additionally, Jhaveri et al. developed Tf-modified resveratrol liposomes (Tf-RES-L) for treating glioblastoma (GBM), which enhanced the anticancer effects of resveratrol, inhibited tumor growth, and extended mouse survival, demonstrating promising therapeutic potential [162].

In further research, Kang et al. developed muscone and TfR-targeted docetaxel (DTX) liposomes, successfully enhancing the drug penetration of the BBB and improving glioma treatment outcomes [163]. RI7217, a mouse monoclonal antibody with high affinity and selectivity for TfR, enables brain-targeting capabilities [163]. Researchers loaded DTX into liposomes and co-modified them with muscone and RI7217 to enhance the targeting specificity. Similarly, Li et al. developed an actively targeted biomimetic liposome system (Tf-ELE/CTX@BLIP) for delivering elemene (ELE) and cabazitaxel (CTX) to treat glioma [164]. This system modifies the liposome surface with Tf and embeds RG2 glioma cell membrane proteins, achieving efficient BBB penetration and tumor-targeted delivery, extending mouse survival, and showing low toxicity.

However, exogenous transferrin may competitively bind with TfR, thus limiting drug delivery efficiency [194]. To overcome this challenge, researchers have proposed various alternative strategies, such as using the small peptide T7 (HAIYPRH) as a targeting ligand. Unlike transferrin, T7 peptide can specifically bind to TfR, enhancing the ability of drugs to penetrate brain tumor cells with its small molecular weight and high targeting specificity [68,157,159]. Wang et al. designed a T7-modified nanomedicine capable of crossing the intact BBB in a mouse model and targeting delivery in intracranial gliomas, utilizing transferrin’s natural binding properties for efficient accumulation in brain tumor regions [189]. Additionally, Mojarad et al. used vincristine (VCR) as a model chemotherapeutic drug to compare various liposomal formulations based on TfR-targeting peptides (including T12, B6, and T7), finding that T7-modified liposomes exhibited superior BBB penetration and brain tissue distribution [165]. This system showed optimal anti-glioma effects in vitro through targeting actions and apoptosis induction mechanisms, providing an effective brain-targeted drug delivery platform.

In summary, TfR serves as a key target for brain glioma and glioblastoma, enabling efficient drug delivery through receptor-mediated transcytosis and significantly enhancing therapeutic effects. Tf-modified nanocarriers combine the advantages of active targeting and drug delivery, offering new insights and potential therapeutic strategies for overcoming brain tumors.

#### 4.1.2. Interleukin-13 Receptor

Gliomas typically exhibit infiltrative growth with indistinct boundaries, making complete resection through traditional surgery challenging [147]. Conventional radiotherapy and chemotherapy also pose significant risks of irreversible damage to normal brain tissue. Consequently, developing more precise targeted therapeutic strategies is of paramount importance. The interleukin-13 receptor (IL-13R) is highly expressed in gliomas but nearly absent in normal brain tissue, making it an ideal therapeutic target [195,196]. IL-13Rα2 not only enhances the invasiveness of glioma cells but also promotes tumor cell proliferation in the presence of the mutant epidermal growth factor receptor (EGFRvIII) [195,197]. The high expression of IL-13R is closely associated with the infiltrative growth of gliomas and their immunosuppressive microenvironment [198]. IL-13R facilitates tumor cell proliferation and invasion by binding with EGFRvIII and activating the RAS/RAF/MEK/ERK and STAT3 signaling pathways [195,198]. Antibodies and antibody–drug conjugates (ADCs) targeting IL-13R have shown effective antitumor activity in animal models, further validating IL-13R as a potential therapeutic target [196,199].

Research has found that the short peptide Pep-1 can cross the BBB and binds with a high affinity to IL-13Rα2 [200]. Based on this, Pep-1 modified nanoparticles have been developed for targeted drug delivery, showing potential in glioma treatment. Borneol, a traditional Chinese medicine, can enhance drug permeability across the BBB by improving the expression of intercellular adhesion molecule-1 (ICAM-1), thereby enhancing drug distribution in the brain [201]. Additionally, its anti-inflammatory and antioxidant properties have made it widely utilized in the treatment of cardiovascular and cerebrovascular diseases [201]. Although borneol enhances the brain targeting of drugs, using borneol-modified nanoparticles alone has limited effects on drug retention in the brain, necessitating a combination with other strategies [202,203]. To address this, Guo and colleagues developed a bifunctional micelle (Pep-1/Bor/CMS-M) aimed at treating gliomas by utilizing Pep-1 to target overexpressed IL-13R and incorporating borneol to enhance the blood–brain barrier penetration [166]. This micelle significantly enhances cytotoxicity and internalization in glioma BT325 cells, increases drug retention in brain tissue, inhibits tumor growth, and extends survival in glioma mouse models, providing new insights for precise glioma treatment. Wang and colleagues developed a PEGylated nanoparticle conjugated with Pep-1 (Pep-NP-PTX) for glioma treatment, demonstrating a significantly enhanced uptake in C6 cells and a lower IC50 in vitro [167]. In vivo studies reveal that Pep-NP-PTX has a significantly higher distribution in glioma tissues compared to unmodified nanoparticles, significantly extending mouse survival and outperforming traditional treatment strategies. Additionally, Lv and colleagues studied a novel paclitaxel-modified nanoparticle (PC-NP-PTX) that combines Pep-1 and CGKRK peptide for the dual-targeted chemotherapy of glioma cells and neovasculature, utilizing a high expression of IL-13Rα2 on glioma cells and heparan sulfate on endothelial cells to achieve a brain-targeted delivery [168]. PC-NP-PTX shows notable advantages in enhancing uptake and antitumor activity in HUVEC and C6 cells. In vivo experiments demonstrate effective accumulation in glioma regions, significantly extending mouse survival time beyond other treatments without apparent acute toxicity.

In summary, the IL-13R-targeted drug delivery system combined with strategies to enhance blood–brain barrier penetration shows great potential in glioma treatment.

#### 4.1.3. Lactoferrin Receptor

The high expression of the lactoferrin receptor (LfR) in the BBB and brain tumors has become a focal point in recent research on brain-targeted drug delivery [204,205]. Lactoferrin (Lf), as a natural ligand, facilitates efficient drug delivery by binding to LfR [205,206]. Multiple studies have demonstrated that modifying drug carriers to bind with LfR can significantly enhance the efficiency of drugs crossing the BBB and improve treatment outcomes for brain diseases.

For instance, Kuo et al. developed a lactoferrin-modified liposome system for delivering quercetin to treat AD [169]. This system significantly improved drug crossing efficiency through the BBB and enhanced cognitive abilities in AD model mice by restoring neuron function. Similarly, Tang and colleagues designed a nanoparticle system co-modified with Lf and borneol (Lf-BNPs) for encapsulating dopamine to treat PD [170]. This system not only enhanced the efficiency of drugs crossing the BBB but also effectively alleviated pathological symptoms in PD model rats by mitigating 6-hydroxydopamine-induced striatal lesions, improving contralateral rotational behavior, and increasing striatal monoamine neurotransmitter levels. Huperzine A, an alkaloid extracted from Huperziaceae plants like Huperzia serrata, is a potent acetylcholinesterase inhibitor with notable applications in treating cognitive impairments [207]. Meng et al. developed a Lf-modified PLGA nanoparticle system for delivering Huperzine A via nasal administration, for the treatment of AD, enhancing brain targeting and demonstrating prolonged drug release [171]. In brain tumor therapy, Qi et al. created a muskone and Lf co-modified liposome system for delivering docetaxel to treat gliomas, showing an improved drug uptake and antitumor efficacy [172].

In conclusion, Lf-modified drug carrier systems show great potential in enhancing the drug crossing efficiency of the BBB, improving targeted delivery, and ameliorating treatment outcomes for CNS diseases. These studies provide crucial theoretical and practical support for the development of brain-targeted drug delivery technologies.

#### 4.1.4. Low-Density Lipoprotein Receptor

The low-density lipoprotein receptor (LDL-R) is a transmembrane protein found predominantly in the liver, endothelial cells, and other metabolically active tissues, playing a crucial role in regulating cholesterol levels by binding low-density lipoproteins (LDL) [208]. In the brain, LDL-R is extensively expressed on brain microvascular endothelial cells (BMECs), where it not only participates in cholesterol metabolism and transport but also serves as a promising target for drug delivery [209]. This prominent expression makes LDL-R a central hub for receptor-mediated transcytosis, facilitating drug carriers to cross the BBB and achieve a precise targeting of brain diseases.

In brain drug delivery research, the interaction between apolipoprotein E (ApoE) and LDL-R strongly supports the drug passage across the BBB. ApoE shows notable LDL-R binding on BMECs, which offers a significant potential for targeted drug delivery [210]. For instance, Kuo and Rajesh developed rosmarinic acid-loaded polymer nanoparticles by combining CRM197 and ApoE for the treatment of AD, significantly enhancing brain delivery efficiency and exhibiting excellent neuroprotective effects in cell and animal studies [173].

Additionally, Yang et al. introduced an innovative pH-responsive active targeting drug delivery system to improve paclitaxel (PTX) effectiveness [174]. This system exploits mitochondria’s critical role in cancer cells, allowing berberine (BBR) to preferentially target mitochondria. By attaching polyethylene glycol (PEG) and folic acid (FA) to liposomes through acid-sensitive hydrazone bonds, the system enhances glioma targeting. Tween 80, combined with ApoE and ApoB, facilitates drug BBB penetration via LDL-R-mediated endocytosis, significantly enhancing treatment efficacy in brain tumor regions [174].

Moreover, Zhang et al. developed a peptide-22 modified dual-targeting paclitaxel nanoparticle, leveraging peptide-22’s specific affinity for LDL-R to assist drug BBB penetration and brain tumor targeting effectively [175]. These nanoparticles accumulate extensively at glioma sites and deliver drugs through caveolae and clathrin-mediated endocytosis, greatly improving the targeting efficacy [175].

Overall, LDL-R and ligand-based drug delivery strategies can overcome BBB limitations through receptor-mediated transcytosis, demonstrating a substantial potential in treating brain diseases such as AD and gliomas. These studies provide new insights into brain drug delivery and bring hope for treating refractory neurological diseases.

#### 4.1.5. Low-Density Lipoprotein Receptor-Related Protein

Low-density lipoprotein receptor-related protein (LRP) is a multi-ligand receptor involved in several physiological processes, including lipoprotein metabolism and extracellular protease homeostasis [211]. LRP plays a crucial role in the pathogenesis of AD, such as mediating the local clearance of the amyloid-β protein by cerebral vascular smooth muscle cells and regulating the transcellular transport of amyloid-β peptides across the BBB [212]. Due to these functions, LRP has become a key molecule for targeting brain drug delivery systems.

Several studies have proposed drug delivery systems targeting LRP to enhance drug accumulation and therapeutic effects in the brain. For instance, Sun et al. developed a dual-modified cationic liposome system loaded with PTX and survivin siRNA, combining Angiopep-2 (LRP ligand) and the RNA aptamer A15 targeting CD133, which efficiently crosses the BBB and delivers drugs to CD133 cancer stem cells in gliomas [176]. This system not only enhanced the efficacy of PTX but also significantly extended the survival of model animals by inhibiting cancer stem cell resistance and inducing apoptosis.

Moreover, Song et al. developed a micelle system with Angiopep-2-modified isoliquiritigenin (ISL) to address the poor solubility and low bioavailability of ISL in the treatment of acute ischemic stroke (AIS) [177]. This system significantly improved the drug encapsulation efficiency and brain accumulation, reducing brain injury in a mouse model of acute stroke by inhibiting autophagy and neuronal apoptosis. Zhang et al. introduced a novel nanoparticle system loaded with salidroside (Sal) and icariin (Ica), targeting AD using Angiopep-2 [178]. The liposomes exhibited excellent drug delivery properties, increasing brain drug accumulation, improving cell uptake efficiency, and reversing neurodamage, inhibiting neuroinflammation and oxidative stress, thereby improving cognitive function.

In conclusion, LRP-targeted drug delivery systems show an immense potential in treating brain diseases such as gliomas, acute stroke, and AD. These studies provide evidence that the precise targeting of LRP can significantly enhance brain drug delivery efficiency, improve therapeutic outcomes, and reduce side effects.

#### 4.1.6. Integrin

Integrin receptors are a class of transmembrane glycoproteins composed of α and β subunits, crucially involved in interactions between cells and the extracellular matrix (ECM), and playing a significant role in regulating cell adhesion, migration, and growth [213]. In brain delivery, integrin receptors facilitate the passage of drugs or nanocarriers by specifically binding with endothelial cells of the BBB, enabling targeted therapies in the nervous system [214]. The cyclic RGD peptide, a high-affinity ligand for integrin αvβ3, binds to receptors on brain microvascular endothelial cells and tumor cell surfaces, showing promise in treating drug-resistant brain gliomas [215,216].

Research by Li et al. demonstrates that RGD-modified vincristine–tetrandrine liposomes significantly enhance the targeted delivery to brain gliomas through receptor-mediated endocytosis, reversing MDR by inhibiting P-gp protein expression [181]. These liposomes also induce programmed cell death in glioma cells by activating apoptotic signaling pathways like caspase-8, caspase-9, and caspase-3, effectively extending the survival of tumor-bearing mice. Wang et al. developed a solid lipid nanoparticle system modified with a cyclic RGD peptide for delivering paclitaxel and naringenin in the treatment of polymorphic GBM [182]. This system improved drug encapsulation efficiency and release rates, enhancing pharmacokinetic properties in vivo. In vitro studies show that SLNs have a higher cytotoxicity in U87MG glioma cells compared to free drugs, and dye-labeled SLNs demonstrate a better uptake efficiency than conventional solutions, indicating superior anticancer activity in GBM treatment.

To address challenges in glioblastoma drug delivery, particularly the BBTB and high interstitial fluid pressure, Gu et al. created an innovative drug delivery system (DDS) [183]. This system incorporates the high-affinity MT1-AF7p peptide onto polyethylene glycol-polylactic acid nanoparticles loaded with PTX, enhancing their targeting capability towards glioma cells and neovasculature [183]. Additionally, the research combined the use of iRGD peptide to facilitate the infiltration of nanoparticles from the tumor vasculature into the tumor parenchyma. The iRGD peptide enhances the drug permeability and accumulation in tumor tissues by binding to the overexpressed αv integrin and neuropilin-1 on tumor blood vessels and glioma cells. The dual-targeted DDS strategy, incorporating iRGD peptide, significantly increases the antitumor activity of the drug and extends the survival of mouse models, providing a new solution for effective glioblastoma treatment.

Moreover, the pH-responsive active targeting drug delivery system (PTX-TR-Lip) developed by Shi et al. enhances paclitaxel efficacy by optimizing transport mechanisms [184]. This system exploits the high affinity of TR peptide for integrin αvβ3, improving targeting while possessing pH-responsive cell-penetrating characteristics. These liposomes demonstrate strong BBB crossing ability, effectively targeting glioma cells and brain cancer stem cells, and disrupting vascular mimicry channels.

In summary, cyclic RGD peptide-modified drug delivery systems significantly improve the targeting, efficacy, and bioavailability in brain glioma treatment, providing effective solutions to overcome the blood–brain barrier and tumor resistance.

#### 4.1.7. Nicotinic Acetylcholine Receptor

Nicotinic acetylcholine receptors (nAChRs) are ion-gated receptors expressed in neurons and brain microvascular endothelial cells, playing a crucial role in regulating the neural signal transmission by interacting with ligands such as acetylcholine or nicotine [185,217,218]. Upon activation, they allow the permeation of cations (e.g., Na^+^, K^+^, and Ca^2+^), triggering membrane depolarization and participating in higher brain functions like cognition, learning, and memory [219,220,221,222]. The primary subtypes in the brain include α4β2, which modulates synaptic transmission, and α7, which is associated with Ca^2+^ permeability and neuroinflammation [223,224]. Notably, nAChRs play a pivotal role in neurodegenerative diseases, where the α7 subtype interacts with β-amyloid (Aβ), exacerbating neurotoxicity and memory deficits [225,226]. Additionally, nAChRs serve as key targets for drug development, including acetylcholinesterase inhibitors for treating AD and partial agonists like varenicline for smoking cessation, which improve symptoms by regulating receptor activity [227,228,229]. Thus, nAChRs’ extensive roles and adaptability position them as crucial targets in studying therapies for cognitive deficits and addiction-related diseases [225,226].

In brain-targeted drug delivery, the rabies virus glycoprotein peptide (RVG29) has been identified as capable of binding to nAChRs and crossing the BBB, offering novel solutions for delivering drugs to the brain [230,231,232]. Xin et al. developed an RVG15-modified liposomal system (RVG15-Lipo) for delivering paclitaxel to gliomas [185]. This system enhanced BBB penetration through high-affinity nAChR binding and significantly suppressed the tumor growth in glioma mouse models. Compared to RVG29, RVG15 exhibited a superior delivery efficiency due to its lower molecular weight, reduced costs, and smaller particle size, presenting a more efficient option for brain-targeted drug delivery [185,217].

Additionally, Li et al. designed an RVG29 peptide-modified BA-PEG-PLGA nanoparticle system for the intranasal delivery of baicalin (BA) to treat cerebral ischemia [186]. This system demonstrated stable drug release improved bioavailability and reduced peripheral drug distribution. Studies showed that intranasal administration significantly improved neurological deficits, reduced infarct size, and alleviated neuronal damage in ischemic rat models by activating the Nrf2/HO-1 pathway and suppressing inflammatory cytokines.

Further advancements by Zou et al. introduced a hybrid nanoparticle system with a mixed lipid monolayer, biodegradable polymer core, and RVG peptide for a paclitaxel delivery to malignant gliomas [187]. This system demonstrated efficient BBB penetration, a selective targeting of tumor-associated macrophages (TAMs), controlled release, and tumor-specific toxicity, exhibiting a strong anti-glioma efficacy in mouse models and providing a promising approach for brain-TAM-targeted therapies.

#### 4.1.8. Nucleolin

Nucleolin is a protein overexpressed in cancer cells such as gliomas, present on the tumor cell membrane and recognized by specific ligands, serving as an important target for drug delivery [233,234,235]. Studies have revealed that the aptamer AS1411, derived from single-stranded DNA, can bind with a high affinity to nucleolin on glioma cell membranes, making it a promising system for targeted drug delivery [236]. Shikonin (SKN), obtained from the Lithospermum herb, induces apoptosis in tumor cells and reduces cancer stem cell numbers in gliomas by inhibiting protein tyrosine kinase activity and affecting tumor angiogenesis, thus enhancing drug sensitivity [237,238]. However, due to SKN’s poor water solubility, its absorption in vivo is limited, necessitating a formulation optimization for improved targeted cellular internalization. Wang et al. developed an AS1411 aptamer-modified microemulsion system (AS1411/SKN&DTX-M), co-loaded with SKN and docetaxel, utilizing nucleolin’s high expression for targeted drug delivery [188]. This system can effectively cross the BBB and efficiently accumulate at glioma sites. Its mechanism mainly involves the high-affinity binding of AS1411 to nucleolin, enhancing drug targeting in the brain and significantly inhibiting glioma growth. Another study by them reported a magnetic T7 peptide and AS1411 aptamer-modified microemulsion (Fe_3_O_4_@T7/AS1411/DTX&SKN-M), which combines tumor-targeting ligands and ultrasmall superparamagnetic iron oxide nanoparticles [189]. This system accumulates in the brain under an external magnetic field, distributing within gliomas via the affinity for nucleolin/transferrin receptors, thus delaying tumor growth. Additionally, research indicates that nucleolin is closely related to the regulation of the brain tumor immune microenvironment, suggesting that a further exploration of its mechanisms could offer new insights for developing more precise drug delivery systems [239].

In conclusion, RMT technology effectively overcomes the BBB by utilizing specific receptor binding, offering new hope for treating brain diseases. From TfR to LfR to LDL-R, research and application across these targets demonstrate the significant potential of this technology in precise brain-targeted drug delivery. Particularly in treating gliomas, neurodegenerative diseases, and other challenging brain diseases, RMT provides viable breakthrough solutions. Despite challenges like receptor saturation and nonspecific binding, continuous technological optimization ensures broad future application prospects for RMT in brain drug delivery, promising more precise and efficient clinical treatment options.

### 4.2. Carrier-Mediated Transport

Carrier-mediated transport (CMT) is a key mechanism for natural products to cross the BBB, relying on the high expression of endogenous transport proteins in brain microvascular endothelial cells, such as glucose transporter (GLUT1) and amino acid transporter (LAT1), which transport essential nutrients from the blood to brain tissues [240,241]. The core of the CMT mechanism involves the interaction of drugs or carrier molecules with these endogenous transport proteins, mimicking the structure or function of endogenous substrates to achieve efficient drug transport [28,241,242]. Hence, leveraging the carrier-mediated transport mechanism offers new design concepts for developing brain-targeted drugs from natural products, particularly in the areas of small molecule modification and structural optimization. The applications of natural products for brain delivery via the CMT pathway are summarized in Table 2.

#### 4.2.1. Glucose Transporters

The high affinity between transport proteins and their substrates establishes CMT as one of the most promising methods for facilitating drug delivery to the brain [242]. Glucose transporters (GLUT), located on the surface of brain capillary endothelial cells, are recognized as some of the most efficient systems for delivering glucose across the BBB [256,257]. Given that the brain’s energy demands are predominantly sustained by glucose, GLUT plays a fundamental role in this supply mechanism [258]. Remarkably, the glucose transportation efficiency via GLUT is estimated to be 15 to 3000 times higher than that of other transport proteins [244,259]. GLUT1, in particular, stands out due to its high affinity and effective transport capabilities, making it a prime target in the development of brain-targeting drugs. By engineering carriers or drug molecules with glucose moieties or analogous functional groups and utilizing GLUT’s natural transport mechanisms, researchers can significantly enhance the efficiency of drug delivery across the BBB through innovative molecular modifications and ligand design [260].

Numerous studies have explored GLUT-mediated drug delivery systems, particularly for targeting brain tumors such as gliomas. Fu et al. developed a glucose-RGD (Glu-RGD)-modified liposome, significantly enhancing PTX delivery through GLUT-mediated endocytosis. Acting as a ligand, Glu-RGD binds to GLUT and integrin αβ receptors, promoting endocytosis. Compared to free paclitaxel, these liposomes increased the in vivo uptake and concentration efficiency by 4.41 and 4.72 times, respectively [244]. Similarly, Zhu et al. designed multifunctional liposomes containing paclitaxel and ginsenoside Rg3 (Rg3-PTX-LPs), which significantly improved drug permeability and brain targeting in both in vitro and in vivo experiments [243]. Rg3, a ginseng-derived compound, interacts with GLUT on cancer cells, improving paclitaxel’s permeability while also replacing cholesterol in liposomes and enhancing drug efficacy [261]. Wang et al. developed a system targeting glycosylated heptapeptide A7R (ATWLPPR), which binds to VEGFR2 and NRP-1 receptors, further enhancing the paclitaxel transport to gliomas [248].

To address the limited BBB penetration of artemether, a potent antitumor agent, Li et al. combined it with paclitaxel in a system utilizing mannose-vitamin E (MAN-TPGS1000) and dimeric ammonium-lipid (DQA-PEG2000-DSPE) derivatives [246]. This strategy leverages GLUT-mediated endocytosis and charge-based interactions to enhance paclitaxel’s bioactivity, inducing apoptosis in brain cancer cells by modulating angiogenesis and pro-apoptotic pathways [246].

In AD research, Barbara et al. developed curcumin-loaded poly(lactic-co-glycolic acid) (PLGA) nanoparticles modified with G7, a glycosylated hexapeptide ligand targeting GLUT-1, a key glucose transporter in the BBB [247]. By mimicking glucose, G7 binds GLUT-1, facilitating cellular internalization via clathrin-mediated endocytosis [262,263,264,265,266]. Similarly, Chen et al. developed glucose-modified quercetin liposomes (QU-Glu-Lip), significantly enhancing the quercetin delivery to the brain through a GLUT1-mediated mechanism. This system reduced oxidative damage and demonstrated neuroprotective effects, highlighting its potential for neurodegenerative disease treatment [252].

In glioma research, Wang et al. designed MAN-modified liposomes that enhanced BBB transport and targeted gliomas, delivering drugs to regions such as the cortex, cerebellum, and hippocampus [249]. Ying et al. improved glioma therapy by incorporating ursolic acid (UA) and epigallocatechin 3-gallate (EGCG) into targeted liposomes [251]. Kong et al. developed a dual-ligand liposome combining MAN and wheat germ agglutinin (WGA) to load epirubicin, significantly enhancing brain targeting and prolonging survival in glioma-bearing rats [250].

Beyond gliomas, GLUT-mediated systems have been applied in cerebral malaria treatment. Tian et al. developed na-ATS/TMP@lipoBX, a liposome system using cholesterol-undecanoic acid-glucose conjugates, targeting GLUT1 to enhance drug delivery and reduce infection recurrence in mice [253].

Vitamin C transport also highlights GLUT’s potential. Dehydroascorbic acid (DHAA), the oxidized form of vitamin C, crosses the BBB via GLUT1, while ascorbic acid (AA) is actively transported by SVCT2, a key vitamin C transporter in the brain [267]. SVCT2, primarily expressed in endothelial cells, choroid plexus epithelial cells, and neurons, facilitates vitamin C accumulation in cerebrospinal fluid and brain tissue, making it a valuable drug delivery target [268,269,270,271,272]. Peng et al. designed a glucose-vitamin C (Glu-Vc) derivative that synergistically utilizes GLUT1 and SVCT2 to enhance paclitaxel delivery to the brain. This system achieved a 7.53-fold higher uptake efficiency and a 7.89-fold higher brain concentration compared to non-modified liposomes, demonstrating a promising vitamin C-based drug delivery strategy [245].

In summary, GLUT plays a crucial role in drug delivery for brain diseases. By harnessing GLUT’s properties, researchers have developed novel systems that enhance brain targeting and hold great promise for treating gliomas, neurodegenerative diseases, and cerebral malaria. These advances provide innovative strategies for future brain disease treatment and underscore GLUT’s potential as a therapeutic target.

#### 4.2.2. L-Type Amino Transporters 1

L-type amino transporters 1 (LAT1) is an L-type amino acid transporter highly expressed in brain microvascular endothelial cells, primarily responsible for the transport of essential amino acids [273]. By modifying drug molecules to mimic LAT1 substrates, the brain-targeting ability of drugs can be significantly enhanced [274]. Ferulic Acid (FA), as a natural antioxidant, has potential medical applications, particularly in AD treatment research [275]. However, the low permeability and bioavailability of FA across the BBB limits its clinical application. Puris et al. successfully delivered ferulic acid into the mouse brain using an LAT1-mediated prodrug delivery method, demonstrating a strong barrier-crossing ability and tumor-targeting potential, opening new research avenues for LAT1-mediated drug delivery [254]. Montaser et al. designed and evaluated two LAT1-utilizing derivatives based on probenecid (PRB), which can inhibit MRP and organic anion transporters (OATs) [255]. When used in combination with the chemotherapy drug vinblastine (VBL), these derivatives induced apoptosis in cancer cells, significantly enhancing the pro-apoptotic effect of VBL, outperforming the use of PRB alone. Additionally, these compounds exhibited a good blood compatibility and a low toxicity in healthy cells. The most promising LAT1-targeting inhibitors significantly increased the permeability of VBL across the rat blood–brain barrier, doubling its brain uptake rate. In the in situ rat brain perfusion and intraperitoneal administration in mouse models, these compounds demonstrated an excellent brain-targeting ability and antitumor potential [255]. Therefore, LAT1-targeting inhibitors are considered promising candidates for improving brain-targeted anticancer drug delivery and overcoming MDR issues, providing an important basis for further research.

In summary, LAT1-mediated drug delivery systems have a significant brain-targeting potential, able to overcome BBB limitations and enhance drug uptake in brain tissue, showing broad application prospects.

### 4.3. Adsorptive-Mediated Transcytosis

Adsorptive-mediated transcytosis (AMT) is a drug delivery mechanism that leverages electrostatic interactions between drugs and electronegative components on the BBB surface, such as glycosaminoglycans [264]. Unlike CMT and RMT, AMT does not rely on specific ligand binding, offering unique advantages for delivering positively charged natural products or modified drug carriers [264]. This provides an effective supplementary route for macromolecules or charged molecules to cross the BBB. AMT enhances drug adsorption and transmembrane transport through interactions with negatively charged cell membranes, facilitating an efficient brain delivery of cationic peptides or positively charged carriers. The applications of natural products for brain delivery via the AMT pathway are summarized in Table 3.

Li et al. developed a multifunctional liposome targeting vincristine and tetrandrine for treating brain glioma and eradicating glioma stem cells (GSCs) [276]. This system uses polyethyleneimine (PEI) surface modification to enhance BBB penetration through the AMT mechanism, achieving targeted effects on glioma cells and GSCs via D-α-tocopheryl polyethylene glycol 1000 succinate (TPGS1000-VAP). Vincristine acts by inhibiting microtubule formation, while tetrandrine reverses MDR by blocking P-gp protein expression in both the BBB and tumor cells [276]. This targeted liposome significantly increases the drug accumulation in the brain, enhances the uptake by tumor cells and GSCs, and induces apoptosis by activating pathways like CytC and caspase-3/8/9, thereby extending the survival of tumor-bearing mice.

Additionally, Xiao et al. constructed a WGA-modified vinblastine cationic liposome for brain glioma treatment via the AMT mechanism [277]. This liposome enhances BBB penetration and targeting through WGA surface modification, using DC-Chol as a cationic material to encapsulate vinblastine in the aqueous core, thereby inhibiting tumor metastasis and killing tumor cells. In vitro experiments showed that the targeted liposome effectively induced apoptosis in C6 cells, facilitated drug passage through the BBB, inhibited tumor cell metastasis, and enhanced targeting. Furthermore, mechanistic studies revealed that the targeted liposome downregulated the expression of PI3K, MMP-2, MMP-9, and FAK, thereby inhibiting tumor metastasis. In vivo experiments further confirmed that the targeted liposome exhibited significant antitumor effects at tumor sites and low toxicity to the blood system and major organs, providing a safe and effective treatment strategy for glioma [277].

Compared to other transport mechanisms, AMT’s lack of requirement for specific ligand binding provides a greater flexibility in delivering large molecules or special drugs across the BBB. By incorporating cationic peptides or positively charged modified carriers, AMT can effectively promote drug translocation across the BBB and synergize with other mechanisms. In the future, AMT combined with other targeted delivery technologies, such as magnetic targeting or functionalized liposomes, is expected to further enhance therapeutic efficacy, providing important research directions and breakthrough solutions for drug development and clinical applications in central nervous system diseases.

### 4.4. Other Emerging Delivery Pathways

Beyond the traditional mechanisms of CMT, RMT, and AMT, new emerging delivery strategies are steadily rising, as detailed in Table 4. The ongoing use of techniques like cell-penetrating peptides (CPPs) and cell-mediated drug delivery is fostering significant advancements and innovations in CNS drug delivery.

#### 4.4.1. Cell-Penetrating Peptides

CPPs are a class of short peptides that interact with the anionic components of the BBB through positively charged amino acid residues, such as arginine and lysine, allowing them to rapidly penetrate lipid membranes, bypass the BBB, and deliver drugs to the brain, often referred to as “Trojan Horse” peptides [285]. However, the nonspecific penetrability of CPPs may lead to nonspecific drug distribution in normal brain tissue after systemic administration, raising toxicity concerns [285,286]. Therefore, designing multifunctional tandem peptides by incorporating selective domains or specific targeting ligands has become a key strategy to address this issue.

For example, Liu et al. designed a multifunctional peptide R8-RGD by conjugating the specific ligand cyclic RGD peptide with the cell-penetrating peptide R8 [179]. Compared to R8 or RGD alone, this tandem peptide significantly enhanced liposome penetration into glioma spheroids and BBB models in vitro, and achieved targeted drug delivery to glioma lesions in mice. R8-RGD-modified liposomes loaded with paclitaxel effectively inhibited C6 glioma cell proliferation and induced apoptosis, ultimately extending the survival of tumor-bearing mice. Additionally, this multifunctional peptide exhibits various functions, including BBB transport, glioma targeting, and deep tumor penetration, offering a new approach for the precise treatment of brain glioma.

The nonspecific targeting issue of CPPs has also prompted researchers to explore enhancing drug delivery selectivity through receptor-mediated mechanisms. The R8 domain enhances drug translocation across the BBB and deep penetration within tumors due to its high penetrability. Liu et al. further studied the effects of R8-RGD-modified liposomes on brain cancer stem cells and vasculogenic mimicry, finding that the liposomes that effectively targeted brain cancer stem cells (BCSC) inhibited vasculogenic mimicry (VM) channel formation, and significantly extended the survival in brain glioma mice, with a good biocompatibility and low toxicity [180].

Chen et al. further developed a dual-ligand modified liposome system by conjugating the cell-penetrating peptide TAT and transferrin to drug-loaded liposomes for the co-delivery of doxorubicin and paclitaxel [160]. This system achieved an efficient BBB penetration and deep glioma targeting through the synergistic action of TAT and Tf. In vivo results showed that dual-ligand-modified liposomes significantly prolonged the survival and reduced tumor recurrence risk in a mouse brain glioma model.

In summary, multifunctional tandem peptides combining CPPs with specific ligands or selective domains offer new directions for the treatment of CNS diseases such as brain glioma. Optimizing the liposome surface modification to achieve a targeted and efficient drug delivery is expected to overcome current bottlenecks in brain-targeted therapies, providing safer and more effective treatment options for brain glioma and other neurological diseases.

#### 4.4.2. Cell-Mediated Drug Delivery

Cell-mediated drug delivery is an emerging precision therapeutic strategy that leverages the natural properties of cells within the body to efficiently deliver drugs to specific lesion sites [287]. Compared to traditional drug delivery methods such as direct injection or nanoparticles, cell-mediated delivery offers unique advantages. The core concepts of this strategy can be figuratively described as the “hitchhiking” and “Trojan Horse” mechanisms [288,289]. “Hitchhiking” refers to drugs binding to or being loaded onto carrier cells (such as neutrophils, macrophages, or stem cells), leveraging these cells’ natural migratory abilities and tissue penetration properties to cross the BBB and reach target areas. “Trojan Horse” describes how drugs are protected by carrier cells during delivery, avoiding recognition or premature clearance by the immune system, and once reaching the lesion site, the drugs are released to exert therapeutic effects [289,290,291]. This strategy is particularly suitable for the treatment of brain diseases, such as brain tumors, neurodegenerative diseases, and ischemic stroke, offering a novel solution for drug delivery with its high targeting specificity and low toxicity [292].

##### Mesenchymal Stem Cells

In recent years, mesenchymal stem cells (MSCs) have attracted significant attention as carriers for tumor-targeted therapy. MSCs’ low immunogenicity, rapid ex vivo expansion capability, and inherent tumor-homing properties provide them with a unique advantage in cancer treatment [291]. Not only can MSCs carry therapeutic drugs, but they also effectively migrate to tumor sites, making them especially suitable for treating challenging cancers such as gliomas [293,294]. Their natural tumor-tropism is a crucial feature of MSCs as drug delivery carriers, with tumor tissues secreting cytokines (like TNF-α, IL-6, and SDF-1) that activate MSC-related signaling pathways (such as PI3K/Akt, MAPK, JAK/STAT), inducing their migration to the tumor microenvironment [295]. Thus, MSCs demonstrate great potential for targeted drug delivery.

By combining anticancer drugs with nanoparticles (NPs) and loading them into MSCs, drug-targeted delivery is significantly enhanced. Wang et al. explored the use of paclitaxel (Ptx) encapsulated in PLGA nanoparticles [278]. In their study, Ptx-PLGA nanoparticles were incubated with MSCs, successfully loading the drug into MSCs, and applying it to the treatment of rat orthotopic gliomas. In vitro experiments showed that MSC-loaded Ptx-PLGA nanoparticles greatly enhanced the sustained release of the drug and significantly prolonged the survival of tumor-bearing mice compared to paclitaxel or Ptx-PLGA nanoparticles alone. Moreover, MSCs’ migration ability, cell cycle, and multilineage differentiation potential were almost unaffected, confirming that MSCs can maintain their biological properties while serving as drug carriers [278].

Tian et al. developed an exosome-based targeted delivery system for treating ischemic stroke [282]. They utilized bioorthogonal copper-free click chemistry to conjugate cyclic peptide c(RGDyK) to the surface of MSC-derived exosomes, creating c(RGDyK)-modified engineered exosomes (cRGD-Exo). In a middle cerebral artery occlusion mouse model, an intravenous injection of cRGD-Exo successfully targeted ischemic brain lesion areas and penetrated microglia, neurons, and astrocytes. Loading the natural anti-inflammatory polyphenol curcumin into cRGD-Exo resulted in a significant inhibition of inflammation and apoptosis in lesion areas, outperforming curcumin or exosome treatment alone [282]. This research offers a quick and efficient method for the large-scale production of functionalized exosomes, demonstrating their potential in treating ischemic brain lesions.

In summary, MSCs as a “Trojan Horse” exhibit a significant potential in tumor therapy. By leveraging MSCs’ tumor-homing properties to load nanoparticles like drugs, genes, or diagnostic agents into MSCs, a precise delivery to the tumor core region can be achieved. This approach significantly improves therapeutic targeting, drug distribution uniformity within the tumor, and the retention time compared to traditional nanoparticle delivery methods [296]. Additionally, it reduces drug accumulation in healthy tissues and decreases systemic toxicity [297]. This strategy not only provides an efficient targeted delivery platform for cancer diagnosis and treatment but also opens new possibilities for personalized, multimodal combination therapies such as photothermal therapy, chemotherapy, and gene therapy [298,299,300]. However, further research and optimization are needed regarding MSCs’ long-term safety, delivery efficiency, and interactions with the tumor microenvironment [301].

##### Macrophage

Macrophage-mediated drug delivery systems, as a promising targeted therapy technology, have shown unique advantages, especially in the treatment of brain diseases [302]. Macrophages, an essential part of the innate immune system, have significant chemotactic and migratory abilities, enabling them to cross the BBB and accumulate in inflamed or diseased regions [303]. These traits render them ideal candidates for drug delivery carriers. In this system, drugs can be loaded into macrophages through physical or chemical methods or bind to macrophage membranes, achieving a precise brain-targeted delivery via their natural migration pathways [304,305]. Furthermore, macrophages’ immunomodulatory functions allow them to alleviate neuroinflammation and regulate the brain microenvironment while delivering drugs, thus enhancing their therapeutic effects [306].

Against this backdrop, several studies have explored the application of macrophages in the treatment of brain diseases. For instance, Long et al. developed a macrophage membrane-modified baicalin liposome system (MM-BA-LP) for the treatment of cerebral ischemia-reperfusion injury (CIRI) [279]. Compared to unmodified liposome systems (BA-LP), MM-BA-LP significantly enhanced brain targeting, improved pharmacokinetic parameters, and extended the retention time of baicalin in blood. Pharmacodynamic studies indicate that MM-BA-LP can significantly improve neurological deficits, reduce infarct volume, and ameliorate brain pathological states, exhibiting superior neuroprotective effects compared to BA-LP.

Ischemia-reperfusion (I/R) injury induces metabolic oxidative stress and BBB disruption, leading to secondary brain tissue damage [307]. The accumulation of reactive oxygen species (ROS) not only activates mitochondrial-mediated apoptosis but also further damages the BBB by degrading tight junction proteins [283,308]. To address this issue, He et al. developed a multifunctional biomimetic delivery system based on macrophage-derived exosome carriers (Ex-cur), effectively improving the stability of curcumin through co-incubation [283]. Ex-cur utilizes the inflammation-driven targeting properties of exosomes to precisely migrate to ischemic areas. Curcumin’s antioxidant properties help reduce ROS accumulation, alleviate BBB damage, and inhibit mitochondrial-mediated neuronal apoptosis, significantly improving brain I/R injury. This system has shown significant efficacy in treating cerebral ischemia-reperfusion injury, providing important evidence for its clinical application in other neuroprotective therapies.

Overall, macrophage-mediated drug delivery systems not only show a great potential in the targeted treatment of brain diseases but also enhance therapeutic effects through their immunomodulatory and cytoprotective functions, paving new directions for future clinical treatment of neurological disorders.

##### Red Blood Cells

Red blood cells (RBCs), due to their prolonged circulation time and natural biocompatibility, have become an ideal drug delivery carrier [309]. Their long half-life, which can extend to several weeks, makes them particularly suitable for delivering drugs requiring a sustained release, such as anticancer and antibody drugs [309,310]. Additionally, RBCs can be effectively loaded with drugs on their inner and outer surfaces through chemical modification or physical methods, and can partially evade immune system recognition and clearance, thus reducing the risk of drug-induced immune reactions, enhancing drug efficacy, and minimizing side effects [310]. Despite the significant advantages of RBCs drug delivery systems in prolonging drug action and reducing side effects, the precise control of drug loading and release remains a pressing issue [311]. Relevant studies have shown that appropriate modifications to RBCs membranes can significantly enhance drug delivery efficiency. For example, Han et al. developed a dual-modified biomimetic nanosystem (RVG/TPP-RSV NPs@RBCm) for treating ROS-induced mitochondrial dysfunction-related AD [281]. This system achieves a targeted drug delivery to neuronal mitochondria by loading antioxidants into nanoscale lipid carriers enveloped in red blood cell membranes modified with RVG29 and triphenylphosphonium cation (TPP) molecules. Studies have demonstrated that this modified red blood cell system can significantly enhance the ability of drugs to cross the blood–brain barrier and effectively reduce mitochondrial oxidative stress, improving mitochondrial function, thereby significantly ameliorating cognitive dysfunction in APP/PS1 mice.

##### Neutrophils

Neutrophil-mediated drug delivery systems, as an emerging targeted therapy strategy, have demonstrated a significant potential in the treatment of brain diseases. Neutrophils (NEs), a key component of the human immune system, possess natural BBB penetration abilities and strong inflammation-targeting properties, making them ideal drug carriers [312]. Utilizing the natural migration pathways and targeting capabilities of NEs, drugs can be attached to neutrophil membranes or fused with them through nanotechnology, achieving an efficient delivery to brain lesion areas [313]. Additionally, the immune evasion properties of NEs and their role in alleviating neuroinflammation and oxidative stress in brain pathological processes enhance the applicability of this strategy in treating brain diseases [314].

In the realm of NEs-mediated drug delivery, the Xue team developed a liposome drug delivery system carried by NEs for delivering PTX to inhibit the recurrence of glioma in mice [280]. Studies indicate that NEs can penetrate inflammatory brain tumors and, after tumor resection, move towards inflammatory sites guided by released inflammatory factors. Under the stimulation of concentrated inflammatory signals in the brain, NEs release PTX from liposomes, accurately targeting residual tumor cells. This approach effectively decelerated the tumor recurrence growth and markedly enhanced mouse survival rates, although it did not entirely inhibit tumor regrowth.

Conversely, Tang et al. devised a neutrophil membrane-fused nanoliposome system loaded with leonurine (Leo) for addressing ischemic stroke (IS) [284]. This system considerably improved the drugs’ capability to traverse the blood–brain barrier and significantly ameliorated brain injury in transient middle cerebral artery occlusion (tMCAO) rat models by alleviating neuronal apoptosis, curbing oxidative stress and neuroinflammation, and restoring BBB integrity. The study unveiled the crucial design factors of biomimetic nanosystems in boosting targeting and treatment efficacy, offering new strategies for the targeted treatment of ischemic stroke and establishing a foundation for the extensive medical use of biomimetic nanotechnology.

Overall, emerging delivery systems exhibit significant advantages in delivering natural compounds, particularly in enhancing drug stability, bioavailability, and targeting. Cell-mediated drug delivery, with its unique biological characteristics, demonstrates a great potential in precision medicine and personalized therapy. With the continuous advancements in cell engineering technologies, drug loading strategies, and delivery mechanisms, this technology is expected to further revolutionize medical treatments, providing innovative solutions for various complex diseases. Meanwhile, immune cell-mediated delivery systems leverage the natural chemotaxis and targeting properties of immune cells to deliver natural compounds to the CNS. However, immune cells crossing the BBB are often accompanied by central nervous system inflammation, which may affect the integrity and permeability of the BBB. Although inflammation increases BBB permeability by activating inflammatory mediators, facilitating the delivery of natural compounds by immune cells, it may also pose potential risks. Therefore, when using immune cell delivery systems, it is necessary to balance efficacy with inflammation risks and optimize delivery strategies to minimize side effects.

## 5. Limitations of Trojan Horse Delivery Strategies

Trojan Horse delivery strategies face several significant limitations that hinder their broader application. Specifically, there are several major challenges:

1. The Biocompatibility of Nanocarriers: The safety of nanomaterials in the body is crucial. Some nanomaterials may trigger toxic or immune responses and could even cause long-term side effects to the human body. Therefore, it is necessary to develop safer and more efficient nanocarriers to ensure their widespread application does not adversely affect patient health.
2. The Precise Control of Drug Release: The rate and timing of the drug release are crucial in disease treatment. Adjusting the drug release process according to the needs of different disease stages and targeted sites has become a current research hotspot. This requires an in-depth understanding of the molecular changes in the disease process and precise control of the carrier’s release behavior.
3. Insufficient Targeting Specificity: Although nanocarriers theoretically have some targeting ability, in practical application, drug delivery may still exhibit “off-target” phenomena. This means drugs may not only concentrate in the lesion area but could also affect healthy tissues, leading to side effects. Enhancing the targeting capability of carriers and ensuring drugs act only at target sites remains a significant research topic.
4. Immune Response and Long-term Toxicity: Some nanocarriers, upon entering the body, may trigger immune responses, especially during long-term treatment, where cumulative effects could lead to potential long-term toxicity. Thus, reducing immune rejection and long-term toxicity remains one of the bottlenecks in current technological development.

Future efforts should focus on integrating dynamic carrier features and combining active and passive targeting strategies to enhance specificity and reduce off-target effects. The design of pathogen-inspired carriers in future medical research offers a significant potential to enhance drug delivery efficiency. By mimicking the characteristics of pathogens, these carriers can integrate into biofilms and enable localized, sustained drug release, overcoming the challenges posed by biofilm resistance mechanisms.

## 6. Conclusions and Prospects

The treatment of CNS diseases has consistently faced numerous challenges, stemming from both the complex pathological mechanisms of the diseases and the limitations posed by the BBB. As a natural protective barrier, the BBB effectively blocks harmful substances from entering the brain, safeguarding the nervous system from external damage. However, this protective function also results in a double-edged sword effect. During treatment, the BBB not only acts as a barrier to harmful substances but also impedes many potential therapeutic drugs, hindering their effective delivery to lesion areas and thus reducing therapeutic efficacy. Natural products, with their unique bioactivity and structural diversity, demonstrate exceptional efficacy in fields such as anti-cancer, antibacterial, antiviral, and antioxidant activities. Particularly in neurological disease treatment, they have become a focal point of research due to their unique mechanisms of action. However, the presence of the BBB limits their application in brain disease treatment. Consequently, overcoming the BBB has become a key challenge in CNS disease treatment.

Despite these challenges, recent scientific advancements have made significant progress in overcoming the BBB, particularly with the emergence of breakthrough strategies, offering new possibilities for CNS disease treatment. This article reviews Trojan Horse strategies that bypass the BBB by replicating biological transport mechanisms, such as RMT, CMT, AMT, and emerging delivery technologies like CPP and cell-mediated drug delivery systems, successfully delivering drugs to the brain. These strategies each have unique advantages and limitations. RMT relies on receptor-mediated endocytosis, offering a high specificity but is constrained by receptor expression levels and saturation effects. CMT uses carrier proteins for transport and is suitable for small-molecule drugs, though it is less effective for complex drug delivery. AMT, on the other hand, utilizes electrostatic interactions between drugs and the surface charges of the BBB, providing some degree of universality but lacking targeting specificity. Emerging technologies like CPPs leverage their efficient transmembrane capabilities to deliver various macromolecules and achieve partial targeting through functionalization, while cell-mediated delivery systems harness the natural transport capabilities of cells to deliver macromolecular drugs and even particles. In the future, integrating the strengths of traditional mechanisms with emerging technologies will be critical for achieving a more efficient and precise drug delivery through multimodal strategies.

Meanwhile, Trojan Horse delivery strategies face significant limitations, including challenges in selecting suitable carriers, mismatches in chemical properties between drugs and carriers, poor biocompatibility, and premature degradation, which may lead to toxicity and reduced therapeutic efficiency. Additionally, issues such as uncontrolled drug release, a low loading capacity, and non-specific targeting result in off-target effects and systemic toxicity. While functionalization and the enhanced permeability and retention (EPR) effect offer potential improvements, their consistency remains inadequate. Addressing these challenges requires the development of biocompatible, stimuli-responsive carriers to enable controlled drug release and improved targeting efficiency.

In addition to biological transport mechanisms, advances in nanotechnology have provided a powerful tool for drug delivery. With the continuous advances in nanotechnology and biomedical engineering, emerging technologies such as focused ultrasound (FUS) [315], nanorobotics [316], light-controlled targeted plasma nanobubbles [317], and magnetic nanoparticle-assisted delivery [318] have shown a great potential in brain drug delivery, offering more opportunities for the realization of precision medicine.

Currently, there are many unresolved mysteries regarding the molecular mechanisms of CNS diseases, especially in neurodegenerative diseases (such as AD and PD) and neuroinflammatory diseases (such as multiple sclerosis). Although we have some understanding of the pathology and biological mechanisms of neurological diseases, the existing knowledge framework still cannot fully explain all clinical phenomena. This knowledge gap limits the design of precise therapies and delays the development of new treatments.

Therefore, future research need focus more on in-depth fundamental research, especially understanding the molecular and cellular mechanisms of CNS diseases and their interactions with the BBB. These fundamental studies will provide theoretical support for the development of emerging therapies, laying the foundation for more precise and effective treatment methods. Meanwhile, with the continuous advancement of biomedical technology, interdisciplinary collaboration will become a significant driving force for innovation in CNS disease treatment.

## Figures and Tables

**Table 1:** A summary of the applications of natural drug monomers via RMT *.

Natural Medicine Monomers	Ligands	Receptors	Nano-Drug Delivery System	Application	Ref.
PTX	Tf	TfR	TF/TAT-PTX/DOX-LP	Glioma	[160]
Vincristine/Tetrandrine	Tf	TfR	Tf modified vincristine plus tetrandrine liposomes	Glioma	[161]
Resveratrol	Tf	TfR	f-PEG-PLA-RES nanoparticles	GBM	[162]
Muscone	RI7217	TfR	Muscone and RI7217 co-modified DTX liposomes	Glioma	[163]
Elemene (ELE)	Tf	TfR	Tf-ELE/CTX@BLIP	Glioma	[164]
Vincristine (VCR)	T12, B6, and T7	TfR	T12/B6/T7-LS/VCR	Glioma	[165]
Borneol	Pep-1	IL-13R	DSPE-PEG-Bor	Glioma	[166]
PTX	Pep-1	Interleukin-13 receptor α2 (IL-13Rα2)	Pep-NP-PTX	Glioma	[167]
PTX	Pep-1	Interleukin-13 receptor α2 (IL-13Rα2)	PTX loaded Pep-1 and CGKRK peptide-modified PEG-PLGA nanoparticle (PC-NP-PTX)	Glioma	[168]
Quercetin (QU)	Lf	LfR	RMP-7-Lf-QU-LS	AD	[169]
Borneol	Lf	LfR	Borneol and lactoferrin co-modified nanoparticles (Lf-BNPs)	PD	[170]
Huperzine A	Lf	LfR	HupA Lf-TMC NPs	AD	[171]
Muscone	Lf	LfR	Lf-LP-Mu-DTX	Glioma	[172]
Rosmarinic acid	Cross-reacting material 197 (CRM197)/ApoE	Diphtheria toxin receptor/LDL-R	CRM197-ApoE-RA-PAAM-CH-PLGA	AD	[173]
Berberine	The attachment of plasma apolipoprotein (ApoE and ApoB) to Tween 80	LDL-R	PTX-Tween 80-BBR + FA-Lip	Glioma	[174]
PTX	Peptide-22	LDL-R	PNP–PTX	Glioma	[175]
PTX	Angiopep-2/A15	LRP/CD133	DP-CLPs-PTX-siRNA nanocomplex	Glioma	[176]
Isoliquiritigenin (ISL)	Angiopep-2	LRP-1	ISL loaded micelle prepared with DSPE-PEG_2000_ as the drug carrier modified with angiopep-2	Ischemic stroke	[177]
Salidroside/Icariin	Angiopep-2	LRP-1	Ang-Sal/Ica-Lip	AD	[178]
PTX	R8-RGD	Integrin receptors expressed on C6 cells	PTX-R8-RGD-lipo	Glioma	[179]
PTX	R8-c(RGD) (R8c(RGD)-Lip)	Integrin αvβ3	PTX-R8-c(RGD)-Lip	Vasculogenic mimicry and cancer ctem cells in malignant glioma	[180]
Vincristine/Tetrandrine	RGD	Integrin αvβ3	RGD-modified vinorel- bine plus tetrandrine liposomes	Glioma	[181]
PTX/Naringenin	Cyclic RGD peptide sequence (Arg-Gly-Asp)	Integrin αvβ3	RGD-modified PTX-NAR loaded SLNs	GBM	[182]
PTX	iRGD	Integrin αvβ3	Co-administration of MT1-AF7p-conjugated PEG-PLA with iRGD	GBM	[183]
PTX	TR peptide	Integrin αvβ3	PTX-TR-Lip	Glioma	[184]
PTX	RVG	nAChR	PTX-cholesterol complex (PTX-CHO)	Glioma	[185]
Baicalin	RVG29 peptide	nAChR	RVG29 peptide-modified BA-PEG-PLGA RNPs	Neuroprotection in cerebral ischemia	[186]
PTX	RVG	nAChR	RVG-PTX-NPs	Glioma	[187]
Shikonin	HA/AS1411	CD44/Nucleolin	AS1411 aptamer/hyaluronic acid-bifunctionalized microemulsion co-loading shikonin and docetaxel	Glioma	[188]
Shikonin	AS1411/T7 (HAIYPRH)	Nucleolin/Tfr	Fe_3_O_4_@T7/AS1411/DTX&SKN-M	Glioma	[189]

* Abbreviations: PTX, paclitaxel; Tf, transferrin; Tfr, transferrin receptor; Lf, lactoferrin; LfR, lactoferrin receptor; PLGA, poly(lactic-co-glycolic acid); GBM, glioblastoma multiforme; IL-13Rα2, interleukin-13 receptor α2; AD, Alzheimer’s disease; PD, Parkinson’s disease; ApoE, apolipoprotein E; ApoB, apolipoprotein B; LDL-R, low-density lipoprotein receptor; LRP, low-density lipoprotein receptor-related protein; nAChR, nicotinic acetylcholine receptor; and RVG, rabies virus glycoprotein.

**Table 2:** A summary of the applications of natural products for brain delivery via CMT *.

Natural Medicine Monomers	Substrate	Transporters	Nano-Drug Delivery System	Application	Ref.
PTX	Multifunctional ginsenoside Rg3-based liposomal system (Rg3-LPs)	GLUT1	Rg3-PTX-LPs	Glioma	[243]
PTX	Glucose-RGD (Glu-RGD)	GLUT_1_ and integrin α_v_ β_3_	PTX-Glu-RGD-Lip	Glioma	[244]
PTX	Glucose-vitamin C (Glu-Vc) derivative	GLUT1/SVCT2	Glu-Vc-PTX-Lip	Glioma?	[245]
PTX/Artemether	MAN	GLUT1	PTX-Artemether-MAN-TPGS_1000_-Lip	Glioma	[246]
Curcumin	Glycopeptide (g7)	GLUT1	g7-PLGA-NPs-Cur	AD	[247]
PTX	Glycosylated A7R derivative	GLUT1	^9^G-A7R-PTX	Glioma	[248]
Curcumin	MAN	GLUT1	Curcumin and quinacrine liposomes modified with MAN	Glioma	[249]
Resveratrol	MAN	GLUT1	Epirubicin plus resveratrol liposomes modified with MAN (MAN-EPI-RES-L	Glioma	[250]
Rrsolic acids (UA)/epigallocatechin 3-gallate (EGCG)	MAN	GLUT1	MAN-Ursolic acids plus EGCG liposomes	Glioma	[251]
Quercetin	Glucose	GLUT1	Glucose-modified QU liposome (QU-Glu-Lip)	Neurodegenerative diseases (NDDs)	[252]
Artemisinin	Cholesterol-undecanoic acid-glucose conjugate	GLUT1	na-ATS/TMP@lipoBX	Cerebral malaria (CM)	[253]
Ferulic acid (FA)	L-phenylalanine	LAT1	L-phenylalanine-amino/ester-Ferulic acid	AD	[254]
Vinblastine	Derivatives of probenecid (PRB)	LAT1	PRB-vinblastine	Improves efflux transporter-related MDR of brain-targeted anti-cancer agents	[255]

* Abbreviations: PTX, paclitaxel; GLUT, glucose transporter; SVCT, sodium-dependent vitamin C transporter; LAT, L-type amino acid transporter; and MAN, p-aminophenyl-α-D-manno-pyranoside.

**Table 3:** A summary of the applications of natural products for brain delivery via AMT.

Natural Medicine Monomers	Electropositive Components	Nano-Drug Delivery System	Application	Ref.
Vincristine/ tetrandrine	Polyethylenimine (PEI)/ Vapreotide (VAP)	Vinorelbine plus tetrandrine liposomes modified with PEI and VAP	Glioma stem cells (GSCs)	[276]
Vinorelbine	DC-Chol	WGA (wheat germ agglutinin)-modified vinorelbine cationic liposomes	Glioma	[277]

**Table 4:** A summary of the applications of natural products for brain delivery via emerging delivery strategies *.

Natural Medicine Monomers	Ligands	Nano-Drug Delivery System	Application	Ref.
PTX	R8-RGD	PTX-R8-RGD-lipo	Glioma	[179]
PTX	R8-c(RGD) (R8c(RGD)-Lip)	PTX-R8-c(RGD)-Lip	Vasculogenic mimicry and cancer ctem cells in malignant glioma	[180]
PTX	MSCs	Ptx-encapsulated PLGA nanoparticles (NPs)	Glioma	[278]
Baicalin	Macrophage membrane	Macrophage membrane-modified BA-LP (MM-BA-LP)	Cerebral ischemia reperfusion injury	[279]
PTX	Neutrophils (NEs)	PTX-NEs	Penetrates the brain and suppress the recurrence of glioma	[280]
Resveratrol	Red blood cell	RVG/TPP NPs@RBCm	AD	[281]
Curcumin	MSCs	cRGD-Exo-cur	IS	[282]
Curcumin	Macrophage RAW264.7 cells	Ex-cur	Alleviates cerebral ischemia-reperfusion injury	[283]
Leonurine	Neutrophil membrane	Leo@NM-Lipo	IS	[284]

* Abbreviations: PTX, paclitaxel; MSCs, mesenchymal stem cells; and IS, ischemic stroke.
